# A textual dataset of de-identified health records in Spanish and Catalan for medical entity recognition and anonymization

**DOI:** 10.1038/s41597-025-05320-1

**Published:** 2025-07-01

**Authors:** Salvador Lima-López, Eulàlia Farré-Maduell, Luis Gasco, Jan Rodríguez-Miret, Santiago Frid, Xavier Pastor, Xavier Borrat, Martin Krallinger

**Affiliations:** 1 NLP for Biomedical Information Analysis Unit, Barcelona Supercomputing Center, Barcelona, 08034 Spain; 2 Clinical Informatics, Hospital Clinic, Barcelona, 08036 Spain; 3 Faculty of Medicine, University of Barcelona, Barcelona, 08036 Spain; 4 Grup de Biomarcadors d’imatge multimodal, FCRB-IDIBAPS, Barcelona, 08036 Spain

**Keywords:** Data mining, Medical research

## Abstract

The advancement of clinical natural language processing systems is crucial to exploit the wealth of textual data contained in medical records. Diverse data sources are required in different languages and from different sites to represent global health services. To this end, we have released CARMEN-I, a corpus of anonymized clinical records from the Hospital Clinic of Barcelona written during the COVID-19 pandemic spanning a period of two years. In addition to COVID-19 cases of adult patients, CARMEN-I features multiple comorbidities such as cardiovascular conditions, oncology treatments, post-transplant complications, and infectious diseases. This resource is publicly accessible together with detailed annotation guidelines and granular text-bound annotations generated in a collaborative effort between clinicians, linguists, and engineers to enable training and evaluation of automatic anonymization systems. Moreover, for information extraction purposes, a subset of 500 records is annotated with six relevant clinical concept classes: diseases, symptoms, procedures, medications, pathogens and humans.

## Background & Summary

There is a pressing need to enable access to annotated electronic health records (EHRs) for the development and evaluation of clinical natural language processing (NLP) resources, with the aim to implement de-identification tools and to leverage medical variables of interest that will ultimately advance the use of artificial intelligence (AI) in health services and medical research. This is particularly true for non-English EHR, where only a limited number of resources have been published. Due to the large number of hospital data generated in Spanish-speaking countries and the potential of adapting NLP technologies originally developed for content in Spanish to other romance languages (with more than 900 million native speakers), the release of clinical records in Spanish is now essential.

In the past few years, the global overburden of health systems during the COVID-19 pandemic highlighted the need to develop the implementation of clinical NLP tools for real-world medical data. To this end, a research center and a public hospital, the Barcelona Supercomputing Center (BSC) and the Hospital Clínic de Barcelona (HCB), a tertiary university hospital located in the center of Barcelona, started a collaboration to advance systems capable of processing and analyzing high volumes of unstructured data locked in large collections of clinical narratives. The aim was to produce effective NLP systems to identify patterns, trends, and actionable clinical insights that would remain hidden with the sole analysis of structured data.

CARMEN-I^1^, a corpus of 2,000 de-identified clinical documents of patients admitted with COVID-19 between March 2020 and March 2022, is the result of this collaboration. The corpus contains discharge reports, referral letters, and radiology reports in Spanish and Catalan. The diseases featured are not limited to COVID-19. Coming from a highly specialized hospital, many records also illustrate the severe comorbidities of these patients, such as kidney failure, chronic cardiovascular and respiratory diseases, malignancies, and immunosuppression. The corpus was created with the aim of being a resource for NLP systems and is not intended for clinical research.

The texts in the corpus have been de-identified following a strict, custom-made anonymization protocol that took into consideration Spanish, European, and health services regulations and laws^2,3^ to ensure patient data privacy. The anonymization process involved NLP experts, linguists, and, ultimately, clinicians, who were in charge of validating the final result. Personal Health Information (PHI) contained within the text was manually annotated following a tailored expansion of the MEDDOCAN^4^ anonymization guidelines. Next, two different techniques were applied to the PHI annotations: (1) masking with special tokens, and (2) replacing with synthetic equivalents. Both anonymized versions of the text are released, as well as the annotations for the PHI in each of them.

For training and benchmarking information extraction systems applied to real-world data, CARMEN-I includes semantic annotations of six clinical entity types (diseases, symptoms, procedures, medication, species, and human beings) in a subset of 500 documents. These annotations are based on existing Gold Standard corpora released and validated as part of community-joint efforts known as shared tasks, such as DisTEMIST^5^ or LivingNER^6^. The annotation guidelines for these six entities are made available in Spanish and English. All in all, this layer contains over 26,000 annotations. To validate the PHI and clinical entities layers, basic named entity recognition (NER) transformer models were trained and made publicly available in HuggingFace.

**Fig. 1:**
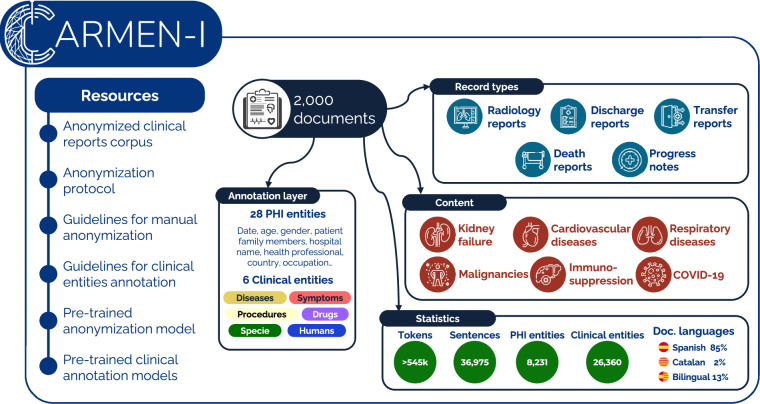
Graphical overview of the CARMEN-I corpus and its associated resources. PHI stands for Personal Health Information.

To the best of our knowledge, CARMEN-I is, at the time of writing, one of the few openly-available corpora comprised of real EHR in Spanish and the first to incorporate text in multiple languages (Spanish and Catalan). Additionally, CARMEN-I incorporates various types of clinical records. Other clinical corpora in Spanish include the Chilean Waiting List corpus^7^, the negation corpus NUBes^8^ or the radiology corpora DisMED^9^ and CARES^10^. Some of the main contributions of CARMEN-I with respect to previous corpora are (a) the release of the PHI annotation layer and a defined anonymization protocol that can be adapted by other institutions who wish to work with EHR in agreement with data privacy laws; (b) the release of semantic annotations that build on and extend pre-existing resources and annotation schemes; and (c) the inclusion of a subset of documents in Catalan or that include code-switching between Spanish and Catalan. Figure 1 presents a summary of the CARMEN-I corpus, its contents, and its associated resources.

CARMEN-I is available on PhysioNet^11^ at https://physionet.org/content/carmen-i/1.0.1/. Due to the sensitive nature of the original data, users must agree to a Data Usage Agreement (DUA) before downloading and using the dataset. We expect to release further versions of the CARMEN dataset incorporating, for instance, the normalization of the annotated clinical concepts to the SNOMED CT ontology.

## Methods

### Data Collection

The clinical reports included in CARMEN-I were generated by health professionals at HCB during the height of the COVID-19 pandemic, between March 2020 and March 2022. The creation of the corpus itself took place between the start of 2021 and mid-2023. To collect the data used in the corpus, raw electronic health records were exported from the Hospital Information System (HIS) in XML format. The extracted files were pre-processed in two steps. First, unnecessary XML tags (such as document metadata and structured sensitive data like document creation date, patient history identifier or patient birth date) were removed. Only elements that were relevant for the interpretation of the contents of the documents, such as section headers, were kept.

To extract the actual text content from the XML, some heavy pre-processing was required in order to fix some artifacts generated by the HIS. This included fixing the document encoding, random spaces inserted between words by the HIS and missing paragraph separations. Finally, discharge and transfer reports, which are especially lengthy, were fragmented based on their sections as indicated by the headers in the XML files. Section headers inform about the contents (e.g. clinical history, physical examination, lab results). By separating these files, we could more easily process them and more importantly, we added hurdles to the re-identification of documents belonging to the same patient.

172,575 documents were extracted in total after separating the reports into sections. To select the final 2,000 documents included in the final version of the corpus, a document selection process was done that involved multiple data selection strategies. These strategies were devised with the aim of creating a representative collection of clinical records that reflected sufficiently enough the diversity of document types across different time ranges and waves of the COVID pandemic. The selection itself was done semi-manually. Following the selection criteria, different features were automatically extracted from the documents to be able to pre-select relevant or interesting documents. Then, a manual revision took manual to evaluate the appropriateness of each document for the corpus. An important part of the feature extraction process was using pre-trained NER models to obtain predictions from the documents for PHI and clinical concepts. The models used are recurrent neural networks with bidirectional LSTM units based on the PharmacoNER Tagger^12^. They were trained using openly-available corpora in Spanish such as MEDDOCAN for PHI^4^, DisTEMIST for diseases^5^ or LivingNER for species and humans^6^. These corpora would later on be also used as the basis of the multiple annotation layers. A look-up system (https://github.com/TeMU-BSC/TEMUNormalizer) was also used to provide preliminary mappings to ICD-10 (the 10th edition of the International Classification of Diseases) and SNOMED CT.

The selection strategies attempted to approach the texts from different perspectives, including textual characteristics, document type and clinical content. In terms of textual characteristics, the number of texts and characters for each text was calculated. This was taken into account to prioritize documents with average length, as well as to revise and include some outlier longer documents if they were deemed relevant. During the last stage of the project, shorter documents were also considered. This was done to alleviate the annotators and clinicians’ workload during the anonymization step and reach the 2,000-document goal, since dealing with shorter documents meant that they were able to work through them faster.

Document types, as well as section types, were another relevant feature. We aimed to have a similar distribution of document types to that provided by the hospital, with discharge and radiology reports being given more priority due to their higher frequency, as well as better pre-processing and clinical information richness. This reflects in the final composition of the corpus, with around 1,800 documents of the 2,000 (90%) coming from these two document types. A hurdle found during this step was the lower quality of progress notes and the very small number of death reports provided by the hospital. Despite this, it was decided to include a few of each of them so that all document types were represented in the final corpus by at least 5 documents. These were manually selected during the last stages of the corpus construction process to make sure that they had sufficiently good pre-processing and that they included some clinical information that was otherwise not present in the already-selected documents.

To drive the selection from a medical perspective, the clinical information contained within the reports was another important criterion. This was a necessary step to make sure that the corpus covered the clinical concepts that the hospital experts deemed relevant during the pandemic. To this aim, a list of pertinent clinical variables was created by some of the physicians treating patients with COVID-19 at the hospital. The diversity of variables encompassed medical history, comorbidities, clinical observations, medications, procedures, lab results and imaging. A small sample of this list can be seen in Table 1. The list of variables was dynamic, since the relevance of some aspects shifted over time as the situation evolved and understanding of COVID-19 increased. For instance, travel history and the administration of chloroquine and azithromycin held significance mainly during the initial months of the pandemic, while vaccination and remdesivir usage increased in importance from 2021 onwards. For this reason, the list was periodically revised and updated by the hospital experts. Within the list, all variables were given an internal ID, a concept class (such as demographic concept, sign or medication), a short description and, if available, an ontology code from ICD-10 and SNOMED CT. The concepts were also enriched with abbreviations and synonyms, both manually by the experts and automatically retrieved from the associated ontologies. Importantly, multiple synonyms in Catalan were added by the experts to improve the coverage in this language. In addition, all entries were classified as either simple or complex. Complex concepts are those that can be decomposed into different concepts. For instance, vasoactive drugs can be decomposed as vassopresors and inotropes, and even further into specific medications such as adrenaline or dobutamine. Finally, concepts already included in the selected documents were marked so that it was clear which were already in the corpus and which were not.

**Table 1:** Sample of the clinical variables list created by the hospital experts used for the document selection step, translated into English.

Concept	Concept Type	Simple/Complex	Description	Synonyms	SNOMED CT Code	ICD-10 Code
Destination of Travel	Contextual	Complex	Locations the patient has traveled to	Travel to (VALUE), Visited (VALUE)	365457007	—
Abnormal Respiratory Rate	Sign	Complex	Finding related to abnormal respiratory rate	Tachypnea, Bradypnea	17077006	R06
Thoracic Pain	Symptom	Simple	Finding related to pain in the chest	Chest pain	29857009	R07.4
Multi-organ Dysfunction	Disorder	Simple	Failure of multiple organ system	Multisystem organ failure	57653000	R68.8
Ventimask	Procedure	Simple	Administration of oxygen with a Venturi mask	VMK	429253002	3E0F7SF

Two different methods were used to try and find the concepts in the list within the clinical records. Firstly, the automatic NER predictions and their mappings were leveraged to identify potential matches for the target concepts. In addition to the NER results, documents with entities that had a low confidence score by the models were extracted since their addition could be useful to reinforce the existing clinical corpora. Secondly, to complement the system predictions and account for the synonyms, spelling variations and concepts in Catalan provided in the clinical variables list, keyword matching algorithms for direct and fuzzy matches were employed to capture additional instances that the first approach might have missed. Again, the output of the systems was revised manually to decide which documents would be chosen. In this step, the output of the PHI NER systems was also quite relevant, as it allowed us to quickly revise and discard documents with specific personal data types that would be hard to anonymize.

The document selection was an incremental process, with documents being selected and shared with annotators and clinicians in batches. The reason for this was two-fold: on the one hand, it allowed to accelerate the entire corpus creation; on the other hand, it also allowed the introduction of more varied documents in the corpus as the hospital generated new reports from the latest COVID waves. The different selection criteria were considered both as a whole and separately during the manual revision, allowing the experts to ensure a diverse representation of documents. This iterative process helped cover a wide range of texts, patient demographics, clinical conditions and treatment variations. The experts were able to identify areas for improvement in the initial batch of documents, as well as repetitive or similar documents, guiding the selection of subsequent documents to ensure that the corpus was not only relevant but also rich in diversity. An important aid during this part of the project was the annotation tool brat^13^, which was used to visualize the documents and see the systems predictions in context.

### Annotation Methodology

CARMEN-I includes two layers of annotation: (1) personal health identifiers (PHI); and (2) clinical entities. All 2,000 documents in the corpus include annotations for the first layer, while the second is only available for a subset of 500 documents, manually selected for their clinical relevance and richness.

For the two annotation layers already-existing, openly-available Gold Standard clinical corpora, validated as part of previous shared tasks, were utilized. The MEDDOCAN corpus^4^ was used for PHI, DisTEMIST for diseases^5^, MedProcNER/ProcTEMIST for procedures^14^, SympTEMIST for symptoms and findings^15^, DrugTEMIST for medications^16^ and LivingNER for species and humans^6^.

These corpora were leveraged to bootstrap the manual annotation process as follows: (a) we used their guidelines as a basis, adding and adjusting concepts to specific characteristics of the hospital records in this setting (e.g. capturing clinical procedures such as *lung CT scan imaging*, *nasal swabs* and *mechanical ventilation* became extremely important); and (b) models trained on these corpora were used to generate suggestions for annotators. These models were iteratively improved by incorporating newly-annotated documents into their training during annotation, a methodology usually known as human-in-the-loop methodology^17^. Finally, to ensure consistency and quality, all documents were manually revised by an experienced annotator who worked on the annotation of the original corpora and post-processed to make sure that all annotations complied with the annotation rules. Other than accelerating the annotation process, one of the main aims of re-using these existing corpora was to extend them. Since the original corpora are made up of clinical case reports, an adaptation of the annotations to actual EHR was necessary to optimize system performance. In this way, the annotations from CARMEN-I complement these corpora and provide a way to fine-tune and evaluate systems for EHR.

**Fig. 2:**
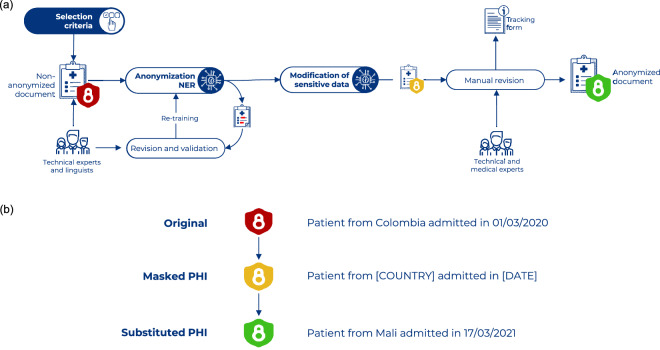
Overview of the anonymization protocol followed for data de-identification. (**a**) Workflow of the data anonymization process. (**b**) Examples of the changes made to the PHI during each step of the anonymization process.

Each of the two annotation layers followed a different workflow. On the one hand, the annotation of sensitive data entailed a multi-step protocol implemented by a team of computational linguists and clinicians. Then, the original annotated data were de-identified using masking and replacement techniques. This protocol is explained in the *Sensitive Data Anonymization* subsection, as well as in Fig. 2. On the other hand, the clinical entity annotation and adaptation of guidelines involved five teams of two physicians led by a trained NLP expert that had worked on the original corpora. This part is described in the *Clinical Entities Annotation* subsection.

#### Sensitive Data Anonymization

The anonymization process consisted of three separate steps: annotation, validation and de-identification. This process is documented in the anonymization protocol, which aims to establish a workflow template and a set of basic criteria to consider a clinical document anonymized, and is released together with the corpus. Figure 2 shows an overview of the different parts of the anonymization process. Side A shows the workflow followed during the process and the different actors involved; side B shows an example of the two different de-identification techniques applied to the data.

The annotation step was accomplished by a team of computational linguists from the Universitat de Barcelona’s Centre de Llenguatge i Computació. The linguists followed the MEDDOCAN corpus guidelines, a document that describes how to annotate sensitive data in clinical documents released as part of the MEDDOCAN shared task^4^. These guidelines, which were originally based on the principles of the United States’ Health Insurance Portability and Accountability Act (HIPAA) and the 2014 i2b2/UTHealth challenge (track 1 on De-identification)^18^ define 28 possible PHI types. The complete list of labels is as follows: FECHAS (dates); EDAD SUJETO ASISTENCIA (patient’s age); SEXO SUJETO ASISTENCIA (patient’s gender); HOSPITAL; FAMILIARES SUJETO ASISTENCIA (patient family mentions: name, age, relationship with patient); NUMERO IDENTIF (ID numbers for specific hospital rooms); NOMBRE PERSONAL SANITARIO (name of health professionals); INSTITUCION (institutions that are not necessarily health-related); PAIS (country); PROFESION (occupation); TERRITORIO (locations other than country names); CENTRO SALUD (health facilities other than hospitals); OTROS SUJETO ASISTENCIA (additional sensitive information about the patient); CALLE (addresses); NUMERO TELEFONO (phone number); ID SUJETO ASISTENCIA (sociodemographic patient identifiers: ethnicity, religion, sexuality); ID CONTACTO ASISTENCIAL (alphanumerical code that identifies the specific episode of contact between patient and health services); and URL WEB.

The MEDDOCAN guidelines were expanded to include hospital and context-specific information such as room names, codes of clinical trials, as well as mentions relevant for the COVID-19 healthcare scenario in Spain such as medicalized hotels. The expanded rules can be found as an annex to the anonymization protocol. In total, the team of computational linguists annotated over 2,000 documents. The annotation was assisted by the predictions of existing PHI detection models to reduce manual annotation efforts. A human-in-the-loop approach was applied to retrain existing models and thus enhance the pre-annotated suggestions. Next, the annotations were revised to ensure anonymization completeness. Throughout the annotation process, each document was reviewed by at least two people to ensure there was no data leakage and to ensure consistency in the annotations.

To further guarantee the absence of direct and indirect PHI, a team of clinicians validated the annotated documents. Here, the clinicians only saw masked versions of the documents in which all personal information was replaced by a generic token that reflected the information type (e.g. “03/03/2020” became “[**FECHAS**]” ([**DATES**]). The clinicians focused on the detection of medical aspects which could potentially identify patients, such as a large number of specific comorbidities, rare conditions or uncommon procedures. Each document was then accepted or rejected based on the criteria laid down in the anonymization protocol. Some controversial aspects were discussed between the different teams until an agreement was reached. In addition, the clinicians classified each document according to its language in one of three classes: Spanish, Catalan or bilingual.

To create the de-identified version of the health records, the annotated PHI were changed using two different approaches:

- **Masking**: Sensitive items are completely removed and replaced by their label name (i.e. hospital names are replaced by the special token “[**HOSPITAL**]”). These items are surrounded by special characters (brackets and asterisks) to distinguish them from normal tokens. Although this de-identification method is easier to implement, the resulting text is visibly anonymized, which might limit its NLP applications. This method is specially useful for health centers that cannot create a fully replaced version of their sensitive text data.
- **Substitution**: Sensitive items are replaced with very similar synthetic substitutes (also called replacements or surrogates). This technique is also sometimes referred as “hiding in plain sight”^19^ and has been used for multiple publicly available biomedical corpora^8,20,21^.
  The substitutes resulting from this technique are created using a combination of gazetteers (lists of words) and rule-based systems. Some of the gazetteers used were scraped from official sources such as Spain’s National Statistics Institute, while others were created manually and included multilingual content to account for the possibility of PHI in either Catalan or Spanish. The rule-based systems were created manually for each annotated PHI type. The approaches for each PHI were originally based on the methodology described for HitzalMed^22^, since both are based on the MEDDOCAN corpus and have a similar labelling scheme. Table 2 provides more details for each of the different substitution functions.
  A potential problem with the semi-automatic nature of this method is that some inconsistencies might arise. For instance, a document might eventually state that an 85-year old person lives with their grandparents. Even then, the texts resulting from this methodology are much more realistic, to the point that it is difficult to discern if they have been substituted or correspond to the original mentions.

**Table 2:** Overview of the substitution functions used to create synthetic replacements for de-identification.

PHI	Gazetteer	Source	Substitution Process
Gender	None	None	This label was kept unchanged.
Name	List of names and surnames	Spanish National Statistics Institute	Items of this class are parsed to separate names and surnames using the gazetteer. They are then replaced from a random element from the corresponding class extracted from the gazetteer. To make this process simpler, surnames are prioritized over names and always returned in case an item is ambiguous or not in the gazetteer.
Age	None	None	Items of this class are parsed using regular expressions. Numerals are then modified by an amount randomly chosen in a [-4, +4] range.
Date	List of weekdays and months’ written form	Manually Created	Items of this class are parsed using regular expressions and detailed heuristics. Days, months and years (if present) are separated and moved by a randomly chosen amount that might be positive or negative. These amounts are the same for all dates in a document so that the document’s timeline is respected. Date calculations are done using Python’s datetime library to maintain temporal coherence.
Occupation	List of occupations classified as job title or job description	MEDDOPROF Occupations Gazetteer^30^	Items of this class are parsed to separate job titles from job descriptions using simple morphosyntactic heuristics. They are then replaced from a random element from the corresponding class extracted from the gazetteer.
Family Member	List of family members classified as ascendant, descendant or neither.	Manually Created	Items of this class are parsed to separate ages (also included within this label) and family relations using simple heuristics. Ages are replaced as explained in the “Age” row. Family relations are parsed to be classified as ascendant, descendant or neither so that an element from the same class might be randomly chosen from the gazettter. Numerals in front of family relations are also randomly replaced.
Clinical Facility	List of made-up and real Spanish clinical facilities of different types	Manually Created	Items are replaced with a random element from the gazetteer. Clinical facility types (e.g. hospital, clinic) are parsed to try and select a replacement of the same type.
City/Country	List of cities and countries with population over 15,000 and geographic features	GeoNames	Items are replaced with a random element from the gazetteer. In case multiple items of the Country and City labels appear together in the text, the geographic features available in the gazetteer (e,g, country code) are leveraged to retrieve geographically-consistent replacements.
Address	List of street names in Donostia/San Sebastián (Gipuzkoa, Spain)	Spanish Government Data Catalogue	Items are replaced with a random element from the gazetteer. A randomly-chosen street number is added at the end.
Numerical ID	None	None	A completely random replacement is created for IDs. For phone numbers, the first digit is unchanged to maintain conventions.
Others	List of manually-decided replacements for each item	Manually Created	Items of the class “OTROS__SUJETO_ASISTENCIA” were replaced manually.

From a more technical perspective, the workflow used for the de-identification process was the following:

1. For each document, the PHI annotations are sorted by their position in the text and processed sequentially. All documents are de-identified independently and the generated replacements are not shared between them.
2. For each sorted annotation, the text before it and after the previous annotation is stored in a variable that will contain the newly de-identified document. The original document is reconstructed incrementally, removing the original PHI and incorporating synthetic replacements in their place. This ensures character offsets between the old and new texts remain aligned, preserving existing PHI annotations. All replaced items are saved in a dictionary so that identical text spans within a document are de-identified with the same replacement. If an annotation already has a replacement, it is used and the next step is skipped.
3. For each new annotation not already-seen by the system, a replacement is created based on its PHI label type. In the masked version, a text replacement is created by placing the label type within brackets and asterisks to create a special token (e.g. “[**NUMERO_TELEFONO**]”. In the substituted version, specific heuristics are used to create a synthetic replacement based on the annotation’s text string. These heuristics are described in Table 2.
4. Once a replacement is generated, it is introduced at the end of the variable containing the de-identified document mentioned in the second step. A new start and end character offsets for the replacement are calculated, and the offsets for all annotations after the current one are adjusted based on the length difference between the original annotation and its replacement. This ensures that all annotations remain aligned with the text. The second step is then repeated until there are no remaining annotations for the current document.

Once all documents had been anonymized and validated, a subset of the data was selected to be annotated with clinical entities.

#### Clinical Entities Annotation

A quarter of the dataset (500 documents) includes annotations for six types of clinical entities. These entities were chosen due to their clinical relevance, as well as the availability of models and guidelines. This allowed to provide annotators with predictions for each document, which were improved utilizing a human-in-the-loop methodology. The re-use of guidelines already validated through an inter-annotator agreement (IAA) phase also sped up the process considerably. The annotated clinical entities (including the Spanish translation for each of the label names, since they correspond to the label names used in the corpus) are:

- **Diseases:** in Spanish, ENFERMEDAD. Diseases are defined as negative deviations from the normal structural or physiological state in any human/animal body. For instance, *broken femur* or *bacterial pneumonia*. This label is based on the DisTEMIST corpus^5^.
- **Symptoms and Findings:** in Spanish, SíNTOMA. This is a broad category which encompasses mainly the complaints and observations of the patient (*pain in the legs*, *agitated*); qualitative results of medical tests (*EKG normal*, *low hemoglobin*); and results of imaging (*nodule in frontal lobe*; *bilateral pulmonary infiltrate*). This label is based on the SympTEMIST corpus^15^.
- **Procedures:** in Spanish, PROCEDIMIENTO. Medical interventions to assist diagnosis and to improve disease, with the exception of names of specific drugs. This category includes interventional radiology (*stent placement*); surgery (*appendectomy*); administration of drug combinations as in chemotherapy regimes; specific diets; rehabilitation; insertion of catethers, etc. It does not include administrative procedures nor clinical scales. This label is based on the MedProcNER/ProcTEMIST corpus^14^.
- **Drugs:** in Spanish, FáRMACO. This category includes exclusively names of single medicines (*paracetamol*; *ivermectin*) and medicines that are usually prescribed in conjunction (*amoxicillin-clavulanate*; *piperacillin-tazobactam*). This label is based on the DrugTEMIST corpus^16^.
- **Species:** in Spanish, ESPECIES. This label includes all living organisms with the exception of human beings, who have their own separate label. Commonly, in these texts we find pathogens (*Staph aureus, Pl. falciparum, Candida albicans*), but also animals (*cat*, *dog*) and food (*fish*, *apples*). This label is based on the LivingNER corpus^6^.
- **Humans:** in Spanish, HUMANO. This category encompasses all mentions of human beings in the text. For instance, health professionals (*nurse*), the patient itself (*patient*), family members of the patient (*mother*, *cousin*), or any other person (*neighbour*, *colleague*). This label is also based on the LivingNER corpus^6^.

The annotation of clinical entities was partly carried out by a team of ten clinicians and healthcare experts. They were distributed in teams of 2 people, according to their specialty. For instance, clinical pharmacologists annotated medicines and clinical microbiologists, species. In addition, an effort was made to provide each team with a different set of documents in accordance with their expertise to maximize the impact of their contribution. For instance, radiologists mainly worked on radiology reports. The teams were managed and supervised by two NLP experts, an expert annotator physician and a computational linguist. During this step, weekly meetings were held where the annotation managers worked together with the clinicians annotating documents and discussing difficult cases. The annotation tool brat’s side-by-side mode was used to compare the different versions of a same document and locate specific disagreements. The clinicians were also provided with documents to work on their own in-between sessions and encouraged to take notes with questions, doubts and suggestions to comment on in future meetings. The role of the clinicians was mostly focused in contributing their clinical knowledge and insights to the already-existing guidelines for each label. This helped cover specific aspects of relevance for the COVID-19 situation, for instance, isolation procedures, new drug combinations, and SARS-CoV-2 diagnostic methods. The resulting annotation guidelines that include all six clinical entities have been published in Zenodo in both Spanish and English.

Due to time constraints, each team only annotated a small subset of the data. Each team annotated a different number of documents based on availability, with some teams annotating as much as 100 documents. In order to obtain a set of 500 documents with all the clinical entities, an in-house annotator (one of the annotation managers), who had also previously participated in the creation of the original corpora, revised the documents annotated by the clinical teams and finalized the annotations. During the initial phases, each team did conduct some parallel annotation rounds as training, which was then used to calculate an initial IAA. Since the scores are not representative of the dataset’s final version, due to the small sample size and the subsequent additions by the in-house annotator, they are not reported here. As an approximate, we report the annotation IAA score for these corpora, which is of 82.30% for diseases, 79.10% for symptoms, 81.20% for clinical procedures, 95.50% for drugs, 94.20% for species and humans.

Before completion, the documents were also post-processed using different heuristics to check the validity and consistency of the annotations. This included, amongst other fixes, locating strings that included unnecessary punctuation or that had been annotated in more than one class. To this purpose, the mentions in the corpus were checked against multiple conditions that were defined based on the annotation guidelines. The output of this step was a list for each condition with possibly problematic annotations. This list was then revised by the in-house annotator, who manually revised that each document to make any relevant corrections.

## Data Records

### Data Description

CARMEN-I^1^ is a collection of 2,000 clinical documents extracted from electronic health records written in Spanish and Catalan. The texts are the product of the first six waves of the COVID-19 pandemic as experienced in one of the main hospitals in Barcelona during a period of two years, from March 2020 to March 2022. All PHI items in the documents have been anonymized via a multi-step de-identification process. The text is presented in two versions: one with masked sensitive data, and another with substituted sensitive data. In addition, the corpus includes annotations for clinical entity recognition (diseases, symptoms, procedures, drugs, species and humans).

From a clinical perspective, the corpus encompasses multiple presentations of COVID-19. Barcelona’s Hospital Clínic is a referral center for complex diseases and organ transplant, which explains why so many patients present severe comorbidities and underlying conditions such as cancer, kidney failure, chronic obstructive pulmonary disease (COPD), cardiovascular diseases and pharmacological immunosuppression.

CARMEN-I includes the following five clinical report types (including the Spanish translation for all report and section types since they are used in the corpus’ file naming scheme):

- **Discharge Reports** (in Spanish, *Informe de Alta* or IA): These reports are created when a patient is discharged from hospital or any other healthcare facility or service. They are quite comprehensive, including the patient’s medical history, current condition, treatment performed during admission, progress, medications prescribed and follow up instructions.
- **Transfer Reports** (in Spanish, *Informe de Traslado* or IT): These reports are created when a patient is transferred between healthcare facilities. They are similar in both content and structure to discharge reports.
- **Death Reports** (in Spanish, *Informe de Exitus* or IE): Death reports contain information similar to discharge reports, with the exclusion of follow up instructions.
- **Radiology Reports** (in Spanish, *Informe de Radiología* or IR): These reports contain imaging results of X-rays, CT scans, MRIs, ultrasounds, angiography, etc. Radiology reports are the most common in the corpus due to their relevance for COVID-19 and because they usually include less sensitive information.
- **Progress Notes** (in Spanish, *Curso Clínico* or CC): These are notes written by doctors and nurses during a patient’s stay. Due to the characteristics of progress notes (longer, repetitive, potentially containing more sensitive data) and pre-processing complexity, only five were included in CARMEN-I.

To facilitate later normalisation and correct use of the data, the following clearly distinctive sections of discharge and transfer reports were processed separately:

- **Medical History** (in Spanish, *Antecedentes*): contains diseases, surgical interventions and relevant life events of the patient.
- **Progress notes** (in Spanish, *Evolución*): notes written by health professionals during hospital admission.
- **Physical Exam** (in Spanish, *Exploración Clínica*): observations about the patient made by the physician, assisted only with instruments like the stethoscope and the reflex hammer.
- **Medical Tests** (in Spanish, *Exploración Complementaria*): measurements obtained with the analysis of bodily specimens (blood, urine, sputum) and of the results of various devices applied on the human body (EKG, EEG).
- **Surgery** (in Spanish, *Intervención Quirúrgica*): intervention within the human body with the assistance of instruments and anesthesia, to remove or correct anatomical elements.
- **Treatment Plan** (in Spanish, *Plan Terapéutico*): scheduled therapy for the patient once they are discharged. For instance, a medicine to be taken twice daily with breakfast and dinner, or respiratory rehabilitation once weekly.
- **Current Problem** (in Spanish, *Proceso Actual*): the reason why the patient is seeking medical attention. For instance, difficulty in breathing or a possible COVID-19 diagnostic.
- **Imaging** (in Spanish, *Radiología*): medical tests that return images within the body, for instance, Magnetic Resonance Imaging.
- **Follow-up** (in Spanish, *Seguimiento*): scheduled visits with one or more health professionals once the patient has been discharged from hospital, for instance, “See cardiologist in 2 weeks.”

The corpus also reflects the multilingual reality of a Catalan hospital. The workforce of this hospital originates mainly from Catalonia, Spain and Latin America. The medical records are written in a combination of Spanish and Catalan, and both languages are often mixed. In this latter case, it is common to see paragraphs and sentences that start in one language and end in the other. A tab-separated value (tsv) file is distributed together with the corpus mapping each document to one of three classes: Spanish, Catalan or bilingual. The bilingual class was applied to documents that incorporate the two levels at any capacity. Naturally, medical expressions, neologisms and accepted acronyms in other languages, in particular but not exclusively English (for example, MRSA for Methicillin-resistant Staphylococcus aureus), have also been recognised and annotated.

CARMEN-I is available to download from PhysioNet^1,11^, a repository of freely-available medical data, operated by the MIT Laboratory for Computational Physiology at https://physionet.org/content/carmen-i/1.0.1/. Users of CARMEN-I must accept a DUA to access the resource. They will also be asked about the intended use of the resource. The purpose of this request is to better know the use of the resource and to inform the patients of the current use of shared data and it will never be used a reason for denying access to the resource. Users must also agree to maintain the anonymization of the data. The resource is under the PhysioNet Contributor Review Health Data License 1.5.0.

The dataset is downloaded as a compressed. zip file that contains three folders, one containing the de-identified text (“txt/”) and the other two containing the stand-off annotations (“ann/” and “tsv”). There is also a tab-separated value file (“CARMEN1_mappings.tsv”) and a “README.md” with a short summary of the corpus, usage and formats. The two versions of the corpus text (“masked” and “replaced”) are located in separate folders. In turn, the annotation folders also contain two more folders for each annotation layer (“anon” and “ner”) The “ann” folder also contains the configuration files to be used in brat. All.txt and.ann files follow the same naming convention: CARMEN-I_{report_type}_section_type_number.extension. For instance: CARMEN-I_IA_ANTECEDENTES_2.txt. Finally, the file “CARMEN1_mappings.tsv” is a tab-separated value file that includes metadata about the corpus files in three columns: “filename”, “language” and “ner_annotations”. The “language” column reflects each report’s assigned language code, which might be “es” (Spanish), “cat” (Catalan) or “bi” (bilingual, contains at least some mixture of both languages). The “ner_annotations” column contains boolean values that indicate whether a document has been annotated wih clinical entities.

The text files are in plain text format (.txt) encoded in UTF-8. The annotations are offered in two formats:

- .ann: This is the annotation tool brat’s standoff annotation format^13^. There is one annotation file for each text file. Each line within the files represents one annotation using five fields: annotation id, label name, start position in characters, end position in characters and annotation text. The first field is separated with the second using a tab; the second, third and fourth fields are separated using whitespaces; finally, the fourth and fifth fields are separated again with a tab. More information on brat’s format is available at https://brat.nlplab.org/standoff.html.
- .tsv: These are tab-separated value files that contain the annotations for each version of the dataset. It contains the following columns: *name* (associated filename), *tag* (annotation label), *span* (start and end character position in text, separated by a comma) and *text* (annotated text string).

## Technical Validation

### Project Documentation

Two important documents have been released together with CARMEN-I: the anonymization protocol (available in Spanish at https://zenodo.org/doi/10.5281/zenodo.10171660and in English at https://zenodo.org/doi/10.5281/zenodo.10171681) and the annotation guidelines for all six clinical entities (available in Spanish at https://zenodo.org/doi/10.5281/zenodo.10171539and in English at https://zenodo.org/doi/10.5281/zenodo.10171646). These documents, created to ensure the consistency and reproducibility of both annotation layers, describe the workflow and decisions taken during the creation of the dataset. They were originally written in Spanish and have been translated into English so that they can be easily re-used by the global community.

On the one hand, the anonymization protocol (shortly introduced in the *Sensitive Data Anonymization* section) guided the different stages of the anonymization process. This document explains the different phases of the anonymization process in depth and also includes, as appendixes, a non-exhaustive list of possible indirect identifiers to be used during the validation step, as well as an extension to the MEDDOCAN annotation guidelines for PHI.

On the other hand, the annotation guidelines for clinical entities are an updated version of the original guidelines used for the corpora the annotation for clinical entities is based on: DisTEMIST^5^ for diseases, SympTEMIST^15^ for symptoms, MedProcNER/ProcTEMIST^14^ for procedures, DrugTEMIST^16^ for medications, and LivingNER^6^ for humans and species. All of these corpora have been used and validated as part of shared tasks (i.e. competitions where various teams develop and benchmark their models against a common dataset to solve a specific problem) and have even become reference corpora used to benchmark clinical systems in Spanish^23–28^.

The CARMEN-I guidelines group the rules for all five corpora within one document for easier access. The rules are divided into different types: general, positive, negative and special. General rules define how to behave in basic situations that apply to all labels, while positive and negative rules describe what must or mustn’t be annotated using a specific label. Special rules are used for situations that are hard to generalize or exceptions to the other rules. Each rule also has a unique identifier, description and definition, as well as illustrative examples. All in all, the guidelines contain a total of 149 rules (17 general, 25 for diseases, 20 for symptoms, 37 for procedures, 13 for drugs, 23 for species and 14 for humans). Other than that, the guidelines also include a short discussion of the CARMEN-I project, basic information about named entity recognition as a task and a description of the different corpora and their associated labels.

### Named Entity Recognition Models

To validate the corpus and give the community the tools to use CARMEN-I in a practical way, we release some named entity recognition models with competitive performance. To further prove the compatibility of the CARMEN-I with the previous datasets it relies on, as well as the application of said datasets in clinical text, we experimented with different combinations of data for training and evaluation. This section describes the architecture and training settings of these models, as well as the different data combinations. All models are made publicly available in HuggingFace (see Model Availability subsection).

#### Architecture and Training Settings

All trained models are based on the pretrained Spanish biomedical RoBERTa model “bsc-bio-ehr-es”, which is available in HuggingFace, and fine-tuned on the different entities. A separate model was trained for each clinical entity (diseases, symptoms and signs, procedures, drugs, species, and humans). In the case of anonymization, it was decided to train a single flat NER model for all of its labels, as there are no nested entities in the whole anonymization corpus.

The training is formulated as a token classification problem, where each token is encoded using the IOB2 schema^29^. Before training, the samples were first split into sentences to limit the input size for the RoBERTa model (maximum 512 tokens). Then, they were pre-tokenized using a regular expression that separates alphanumeric sequences from single non-alphanumeric characters. This separation ensures that punctuation marks adjacent to named entities, like the trailing dot in “The patient presents diabetes.” (where ‘diabetes’ is a Disease entity), are not included in the entity itself, allowing the model to assign more accurate IOB2 labels (e.g., “B-ENFERMEDAD” for “diabetes” and “O” for the dot).

Different sets of hyperparameters were explored for each task to find the best-performing models. Concretely, we tested 20-80 combinations of the following hyperparameters for each training:

- **Batch size:** 8, 16, 32
- **Classifier dropout:** 0.1, 0.3, 0.5, 0.8
- **Learning rate:** 3e-07 to 4e-04
- **Epochs:** 15
- **Warmup ratio:** 0.1
- **Weight decay:** 0.01
- **Weight strategy:** none, frequency, frequency_sqrt

All of these hyperparameters are commonly optimized and adjusted while training neural networks. The only exception is the introduction of our own tag weight strategy, which tries to compensate for the imbalance between IOB2 tags: a large majority of tokens are O tags, whereas some tags like I-NUMERO_TELEFONO only account for 0.002% of tokens. Therefore, we tried different weight strategies for computing the loss.

Finally, each model was evaluated on the validation set after each epoch during training. Only the best epoch of an experiment was used to compare and select the best model. For each task, the model with the highest F1-score on the validation set was chosen. The specific parameters chosen for each model are shown in their respective model card in HuggingFace.

#### Training and Evaluation Data

To validate the CARMEN-I annotated data, as well as to evaluate the impact of using the case reports corpora released for different shared tasks^4–6,14–16^ when applied to real clinical history, we used different combinations of training and evaluation data to train and evaluate the NER models. Three distinct training data configurations were chosen: (a) models trained only on the case reports of the corresponding shared task corpus, (b) models trained only on the CARMEN-I subset for that task, and (c) models trained on the joint case reports plus CARMEN-I.

These three training configurations were then evaluated in two different settings: (i) the corresponding case reports corpus’ test set and (ii) CARMEN-I’s test set. Only the models of training configuration (a), trained on the training split of the case reports corpora, were evaluated using the evaluation setting (i). The reason is that this article focuses on real clinical history and analyzing the usefulness of adding other publicly available corpora to improve the performance on the real data, not the other way around. Furthermore, to maximize the amount of data for the joint models (c), we used the whole case reports corpora (i.e. all splits, including test), making it unfair to evaluate the performance of these models on the case reports (i). All in all, this results in a total of 21 released models, seven for each training data configuration.

For training, validation, and test splits, models with data configuration (a) (that is, trained using only the case reports of the original corpora) used the splits already provided with the corpora. The anonymization corpus MEDDOCAN has splits of 500-250-250 for the three splits, while DisTEMIST (diseases), SympTEMIST (symptoms and signs), MedProcNER/ProcTEMIST (procedures), and DrugTEMIST (drugs) each had splits of 500-250-250 documents. These four corpora share the same set of documents, which are used consistently throughout the three splits. In LivingNER (species and humans), the splits contain 1000-500-485 documents.

For models with data configuration (b) (that is, trained using only CARMEN-I documents), we had to create new splits, which were generated randomly using scikit-learn’s *train_test_split* function. Out of the 500 documents with annotations for clinical entities of CARMEN-I, 300 were used for training, 100 for validation at each epoch during training, and 100 for testing (only for the selected best model). The anonymization layer followed the same process, resulting in 1200-400-400 documents in the three splits.

Finally, for the last data configuration ((c), joint case reports and CARMEN-I case documents), the models were trained on the CARMEN-I training set plus the entire corresponding corpus (that is, including the training, validation, and testing sets). This was done to maximize the diversity and enrich the vocabulary and mentions found.

#### Model Evaluation Results

The performance for the various configurations in the different test sets can be seen in Table 3. Overall, all models and configurations achieved competitive results, with the joint models (c) generally performing best. Results were especially good (over 90% F1-score) on PHI, drug, and human, whereas entities with longer and more complex mentions (symptoms, procedures, and diseases) obtained lower results. These also have more intra-class overlap (i.e., same-class nested entities), which the model cannot produce and thus will fail to detect (favoring only the larger one). On the other hand, all models for species obtained worse results when evaluating on CARMEN-I. This might suggest that LivingNER is easier to learn, perhaps due to the existence of intra-class overlapping annotations and mentions in Catalan in the CARMEN-I species data.

**Table 3:** Evaluation metrics (micro average precision, recall and F1-score) of the baseline models trained on (a) the corresponding case reports (CR) of their shared task (train split), (b) CARMEN-I (train), and (c) CARMEN-I (train) + Case Reports (all: train, validation, test).

Evaluated on	(i) Case Reports (CR)			(ii) CARMEN-I								
Trained on	(a) Case Reports (CR)			(a) Case Reports (CR)			(b) CARMEN-I			(c) CARMEN-I + CR		
	P	R	F1	P	R	F1	P	R	F1	P	R	F1
Anonymization	0.950	0.972	0.961	0.804	0.818	0.811	**0.946**	**0.962**	**0.954**	0.944	0.957	0.950
Drug	0.917	0.909	0.913	0.906	0.885	0.895	**0.954**	**0.928**	**0.941**	0.944	0.926	0.935
Symptom	0.707	0.716	0.711	0.699	0.677	0.688	0.615	0.750	0.676	**0.743**	**0.764**	**0.753**
Procedure	0.757	0.761	0.759	0.746	0.711	0.728	0.746	0.784	0.764	**0.803**	**0.821**	**0.812**
Disease	0.754	0.759	0.757	0.713	0.706	0.709	0.746	0.763	0.754	**0.765**	**0.790**	**0.777**
Human	0.976	0.984	0.980	0.885	0.937	0.910	0.878	0.918	0.897	**0.904**	**0.941**	**0.922**
Species	0.940	0.928	0.934	0.763	0.795	0.779	0.848	0.799	0.823	**0.843**	**0.830**	**0.837**

Evaluation (i) reports the metrics on the test set of the case reports from the shared tasks, while evaluation (ii) reports the metrics on the CARMEN-I test set.

In detail, models trained only on the case reports (a) showed a decreased in performance of 2 to 7 percentage points (pp) when evaluated on CARMEN-I instead of the case reports test set. The drop is even greater for anonymization (15 pp) and species (16.5 pp). When evaluating these models only on the Spanish documents of CARMEN-I (85% of all CARMEN-I), the difference of performance by class is in the range of [-0.4, +1.5] percentage points. Thus, at most, 46.1% of the decrease in performance can be attributed to language (in the case of symptoms), followed by procedures and diseases (above 30%), while the rest have little to no effect or even higher performance on bilingual and Catalan documents, as in the case of anonymization. The remaining decrease of performance (i.e., which cannot be attributed to language) might be due to differences between the domains of CARMEN-I and the case reports, and thus in the mention distributions (new mentions never seen in case reports).

Generally, models trained only on CARMEN-I (b) achieve better results than models trained on case reports (a) when applied to real data, with the only exceptions being symptoms and humans. This highlights the importance of training with real clinical data if the models are to be applied in a clinical environment.

The joint models (c) are overall the best-performing ones on real data, apart from anonymization and drugs, where CARMEN-I-only models (b) already achieved very high results. This suggests that both kinds of datasets are complementary and that case reports are useful in case of real data scarcity, which is often the case. The classes that seem to benefit the most from this additional training data (c) are symptoms and procedures, increasing by 6.5 and 9.4 from case reports-only (a), and 7.7 and 4.8 pp from CARMEN-I-only (b).

Since the anonymization layer contains multiple labels, the anonymization model scores provided in Table 3 are the mean of the results of all individual labels. Table 4 provides a detailed breakdown of the models’ performance for each label. An important detail about the anonymization models is that MEDDOCAN and the CARMEN-I anonymization layer do not share the exact same labels. Due to the high number of labels, which can be very specific, and the low frequency of some of them, there are some classes that only appear in MEDDOCAN and some that only appear in CARMEN-I. To handle this situation in data configuration (c), the labels that only appeared in MEDDOCAN had to be removed.

**Table 4:** Evaluation metrics (micro average precision, recall and F1-score) of the anonymization baseline models trained on (a) the corresponding case reports (CR) of the MEDDOCAN shared task (train), (b) CARMEN-I (train), and (c) CARMEN-I (train) + MEDDOCAN (all).

Evaluated on	(i) Case Reports (CR)			(ii) CARMEN-I								
Trained on	(a) Case Reports (CR)			(a) Case Reports (CR)			(b) CARMEN-I			(c) CARMEN-I + CR		
	P	R	F1	P	R	F1	P	R	F1	P	R	F1
CALLE*	0.810	0.898	0.852	0.098	**1.000**	0.178	**1.000**	**1.000**	**1.000**	0.800	**1.000**	0.889
CENTRO_SALUD*	1.000	0.833	0.909	0.500	0.125	0.200	0.889	**1.000**	0.941	**1.000**	**1.000**	**1.000**
EDAD_SUJETO_ASISTENCIA	0.985	0.990	0.987	0.986	0.948	0.967	**1.000**	**0.993**	**0.997**	0.993	0.980	0.987
FAMILIARES_SUJETO_ASIST	0.630	0.778	0.696	0.609	0.840	0.706	**0.925**	**0.980**	**0.951**	0.839	0.940	0.887
FECHAS	0.990	0.998	0.994	0.848	0.857	0.853	0.955	**0.968**	**0.961**	**0.956**	0.963	0.960
HOSPITAL	0.984	0.931	0.957	0.680	0.576	0.624	**0.894**	**1.000**	**0.944**	0.892	0.983	0.935
ID_CONTACTO_ASIST*	0.884	0.974	0.927	—	—	—	—	—	—	—	—	—
ID_SUJETO_ASIST*	0.956	0.996	0.976	—	—	—	—	—	—	—	—	—
INSTITUCION	0.487	0.552	0.517	0.286	0.273	0.279	0.818	0.818	0.818	**0.950**	**0.864**	**0.905**
NOMBRE_PERS_SANIT	0.978	0.992	0.985	0.857	0.207	0.333	**0.933**	**0.966**	**0.949**	**0.933**	**0.966**	**0.949**
NUMERO_IDENTIF	—	—	—	0.000	0.000	0.000	0.900	0.871	0.885	**0.965**	**0.887**	**0.924**
NUMERO_TELEFONO*	0.926	0.962	0.943	0.600	**1.000**	0.750	0.600	**1.000**	0.750	**0.750**	**1.000**	**0.857**
OTROS_SUJETO_ASIST*	0.000	0.000	0.000	0.000	0.000	0.000	**1.000**	**1.000**	**1.000**	0.600	**1.000**	0.750
PAIS	0.955	0.997	0.976	0.800	0.174	0.286	**0.944**	**0.739**	**0.829**	**0.944**	**0.739**	**0.829**
PROFESION*	0.474	1.000	0.643	0.571	0.444	0.500	**0.824**	**0.778**	**0.800**	0.765	0.722	0.743
SEXO_SUJETO_ASIST	0.991	0.991	0.991	**1.000**	0.977	0.988	**1.000**	**1.000**	**1.000**	0.978	**1.000**	0.989
TERRITORIO*	0.968	0.973	0.970	0.345	0.769	0.476	0.706	**0.923**	0.800	**0.750**	**0.923**	**0.828**
URL_WEB*	—	—	—	—	—	—	—	—	—	—	—	—
CORREO_ELECTRONICO	0.988	0.992	0.990	—	—	—	—	—	—	—	—	—
ID_ASEGURAMIENTO	1.000	0.995	0.997	—	—	—	—	—	—	—	—	—
ID_EMPLEO_PERS_SANIT	—	—	—	—	—	—	—	—	—	—	—	—
ID_TITUL_PERS_SANIT	0.996	1.000	0.998	—	—	—	—	—	—	—	—	—
NOMBRE_SUJETO_ASIST	0.998	0.998	0.998	—	—	—	—	—	—	—	—	—
NUMERO_FAX	0.750	0.857	0.800	—	—	—	—	—	—	—	—	—

Setting (a) reports the metrics on the test set of the case reports, while settings (b) and (c) report the metrics on the CARMEN-I test set. Some labels in the left column are shortened to fit the space. Labels marked with (*) have a support of less than 20 samples in the CARMEN-I test set, while metrics marked with (-) indicate zero samples in the corresponding test set. The labels on the bottom part of the table appear in the MEDDOCAN corpus but not on CARMEN-I.

Another noteworthy characteristic is that some labels in this layer are quite infrequent, resulting in a non-representative evaluation for them. Labels marked with an asterisk (*) in Table 4 had a support of less than 20 samples in the CARMEN-I test split. Even more critically, three labels, namely ID CONTACTO ASISTENCIA, ID SUJETO ASISTENCIA and URL WEB, could not be evaluated at all, as the CARMEN-I test set did not contain any mention for these classes due to the random split and low frequency. Therefore, we cannot properly assess the generalization capabilities of the models for these classes, meaning that they could potentially be overfitting to the few samples of the dataset.

Similarly to clinical entities, in anonymization the performance on the CARMEN-I test set (i.e. evaluation setting (ii)) of both CARMEN-I (b) and joint (c) models are quite high for all labels, while the case reports model (a) only displays good understanding for the most representative classes (i.e., EDAD SUJETO ASISTENCIA, patient’s age, and SEXO SUJETO ASISTENCIA, patient’s sex). The performance drop of the model (a), trained only on case reports, when applied to real clinical data (evaluation setting (ii)) for the remaining classes suggests that the model could not learn to generalize well on these more complex, least-represented classes when provided only with MEDDOCAN data, overfitting to it.

To improve this aspect, zero-shot and few-shot approaches, as well as rule-based models, could be implemented. Furthermore, we could investigate the effect of training a model on case reports only (a) and then sequentially fine-tune it with CARMEN-I data instead of training on the merged datasets, or giving more weight to CARMEN-I samples. This, however, goes beyond the scope of the current article and remains an aspect for future work.

As a final comment, for both anonymization and clinical entities, boundary-relaxed metrics could also be used to evaluate the models. These relaxed metrics consider a prediction correct if there is some overlap with the ground truth mention, which could provide more insight for classes with long mentions. Preliminary analysis during training (on the validation set, without same-class nested entities) show that both symptoms and procedures improve from 79% to 89% on F1-score when using boundary-relaxed metrics instead of strict.

#### Model Availability

All 21 models trained and evaluated in the paper are released to HuggingFace for public use, in an effort towards reproducibility and transparency. They are available as a model collection at Hugging Face (https://huggingface.co/collections/BSC-NLP4BIA/carmen-i-666707832cef2f5b6d51b6ea) and also listed individually in Table 5.

**Table 5:** List of available models in the CARMEN-I HuggingFace collection. The F1 scores reported in this table were calculated with the CARMEN-I test set.

Model Name	Entity	Training Data	F1	URL
bsc-bio-ehr-es-meddocan	PHI	MEDDOCAN	0.811	https://huggingface.co/BSC-NLP4BIA/bsc-bio-ehr-es-meddocan
bsc-bio-ehr-es-carmen-anon	PHI	CARMEN-I	0.954	https://huggingface.co/BSC-NLP4BIA/bsc-bio-ehr-es-carmen-anon
bsc-bio-ehr-es-carmen-meddocan	PHI	MEDDOCAN + CARMEN-I	0.950	https://huggingface.co/BSC-NLP4BIA/bsc-bio-ehr-es-carmen-meddocan
bsc-bio-ehr-es-distemist	Disease	DisTEMIST	0.709	https://huggingface.co/BSC-NLP4BIA/bsc-bio-ehr-es-distemist
bsc-bio-ehr-es-carmen-enfermedad	Disease	CARMEN-I	0.754	https://huggingface.co/BSC-NLP4BIA/bsc-bio-ehr-es-carmen-enfermedad
bsc-bio-ehr-es-carmen-distemist	Disease	DisTEMIST + CARMEN-I	0.777	https://huggingface.co/BSC-NLP4BIA/bsc-bio-ehr-es-carmen-distemist
bsc-bio-ehr-es-drugtemist	Drug	DrugTEMIST	0.895	https://huggingface.co/BSC-NLP4BIA/bsc-bio-ehr-es-drugtemist
bsc-bio-ehr-es-carmen-farmaco	Drug	CARMEN-I	0.941	https://huggingface.co/BSC-NLP4BIA/bsc-bio-ehr-es-carmen-farmaco
bsc-bio-ehr-es-carmen-drugtemist	Drug	DrugTEMIST + CARMEN-I	0.935	https://huggingface.co/BSC-NLP4BIA/bsc-bio-ehr-es-carmen-drugtemist
bsc-bio-ehr-es-livingner-humano	Human	LivingNER	0.910	https://huggingface.co/BSC-NLP4BIA/bsc-bio-ehr-es-livingner-humano
bsc-bio-ehr-es-carmen-humano	Human	CARMEN-I	0.897	https://huggingface.co/BSC-NLP4BIA/bsc-bio-ehr-es-carmen-humano
bsc-bio-ehr-es-carmen-livingner-humano	Human	LivingNER + CARMEN-I	0.922	https://huggingface.co/BSC-NLP4BIA/bsc-bio-ehr-es-carmen-livingner-humano
bsc-bio-ehr-es-medprocner	Procedure	ProcTEMIST	0.728	https://huggingface.co/BSC-NLP4BIA/bsc-bio-ehr-es-medprocner
bsc-bio-ehr-es-carmen-procedimiento	Procedure	CARMEN-I	0.764	https://huggingface.co/BSC-NLP4BIA/bsc-bio-ehr-es-carmen-procedimiento
bsc-bio-ehr-es-carmen-medprocner	Procedure	ProcTEMIST + CARMEN-I	0.812	https://huggingface.co/BSC-NLP4BIA/bsc-bio-ehr-es-carmen-medprocner
bsc-bio-ehr-es-livingner-species	Species	LivingNER	0.779	https://huggingface.co/BSC-NLP4BIA/bsc-bio-ehr-es-livingner-species
bsc-bio-ehr-es-carmen-species	Species	CARMEN-I	0.823	https://huggingface.co/BSC-NLP4BIA/bsc-bio-ehr-es-carmen-species
bsc-bio-ehr-es-carmen-livingner-species	Species	LivingNER + CARMEN-I	0.837	https://huggingface.co/BSC-NLP4BIA/bsc-bio-ehr-es-carmen-livingner-species
bsc-bio-ehr-es-symptemist	Symptom	SympTEMIST	0.688	https://huggingface.co/BSC-NLP4BIA/bsc-bio-ehr-es-symptemist
bsc-bio-ehr-es-carmen-sintoma	Symptom	CARMEN-I	0.676	https://huggingface.co/BSC-NLP4BIA/bsc-bio-ehr-es-carmen-sintoma
bsc-bio-ehr-es-carmen-symptemist	Symptom	SympTEMIST + CARMEN-I	0.753	https://huggingface.co/BSC-NLP4BIA/bsc-bio-ehr-es-carmen-symptemist

For more information on the training and evaluation data of these models, please refer to the Named Entity Recognition Models Section and Table 3. In the table, PHI stands for Personal Health Information.

The models can be used as regular HuggingFace NER/token classification models and can be loaded with the HuggingFace *pipeline* to launch predictions on them. Since the models were trained on pre-tokenized inputs, we recommend conducting the same pre-tokenization during inference. Otherwise, performance might be affected. More information on the model’s license, usage, and metrics is included on each model’s card.

## Usage Notes

CARMEN-I is not suitable for clinical research because specific data related to persons, dates, ages, locations, centers, etc. has been substituted by other values. The dataset is intended to be used for NLP-related purposes, such as model training or benchmarking.

## Data Availability

All annotations described in this article have been done using brat^13^, an open source web-based text annotation tool. Processing and manipulation of the annotated data was performed using the brat_peek Python library (https://github.com/s-lilo/brat-peek), an open source framework to work with brat-annotated data. The open source software spaCy, and more specifically the “es_core_news_lg” model, was used to tokenize and segment documents for the text statistics reported throughout the paper. Custom tokenization rules were incorporated to treat special tokens in the masked versions (e.g. “[**FECHAS**]”) as a single token. The custom scripts and gazetteers used for de-identification will not be publicly released for the moment.
